# Fitz-Hugh-Curtis syndrome

**DOI:** 10.11604/pamj.2022.43.142.30703

**Published:** 2022-11-17

**Authors:** Danilo Coco, Silvana Leanza

**Affiliations:** 1 Department of General Surgery, Ospedali Riuniti Marche Nord, Pesaro, Italy,; 2 Department of General Surgery, Carlo Urbani Hospital, Jesi, Ancona, Italy

**Keywords:** Fitz-Hugh-Curtis syndrome, liver, Italy

## Image in medicine

Fitz-Hugh-Curtis syndrome is characterized by inflammation of the liver capsule and the production of adhesions, leading in pain in the right upper quadrant. It is a rare chronic form of pelvic inflammatory disease that affects women of childbearing age. Curtis described adhesions between the anterior surface of the liver and the abdominal wall in patients with unusual gallbladder episodes during laparotomies in 1930. Similar cases of right upper quadrant abdominal pain were described by Fitz-Hugh, Jr. in 1934. A 40-year-old Caucasian male presented with a progressively abdominal right quadrant pain and left side quadrant pain. He had no medical history but he referred a follow-up for colon diverticulosis from 3 years. He referred satiety and abdominal pain without weight loss fever, nausea and vomiting. Vital signs were within normal limits. Laboratories showed a haemoglobin of 14g/dl, white blood cells (WBC) 8.5x103/ul, platelets 130x103/ul. Liver function tests were normal. CT scan showed a diffuse colon diverticulosis and no signs of cholecystitis or other pathologies. A Clisma Abdomen demonstrated sub-stenosis due to inflammatory diverticulitis. He was referred to operating room. During laparoscopic left colectomy, we found adhesions between the anterior surface of the liver and the abdominal wall violin-string like perihepatic adhesions as Fitz-Hugh-Curtis syndrome that justified the right upper quadrant pain too.

**Figure 1:**
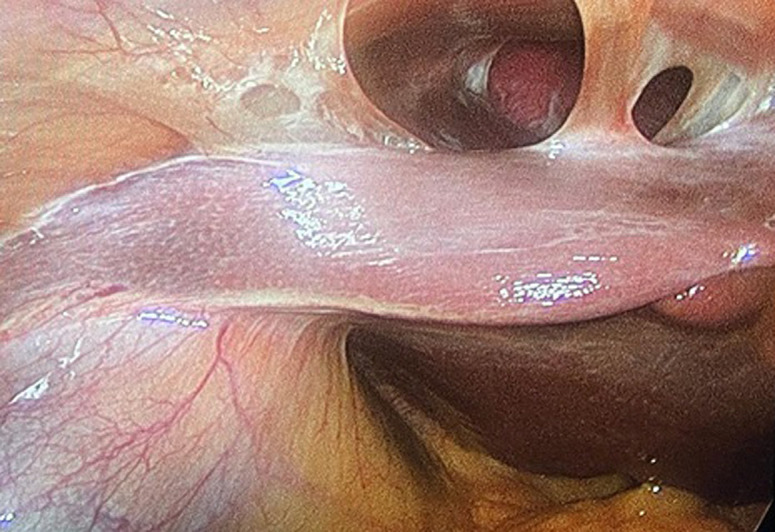
laparoscopic left colectomy showing adhesions between the anterior surface of the liver and the abdominal wall violin-string like perihepatic adhesions as Fitz-Hugh-Curtis syndrome

